# Professorial Advancement Initiative: A Cross-Institutional Collaboration to Increase Faculty Diversity in STEM

**DOI:** 10.3389/fpsyg.2021.733173

**Published:** 2021-10-12

**Authors:** Aman Yadav, Mark J. T. Smith, Charity Rae Farber, Linda J. Mason

**Affiliations:** ^1^College of Education, Michigan State University, East Lansing, MI, United States; ^2^Graduate School, University of Texas at Austin, Austin, TX, United States; ^3^Big Ten Academic Alliance, Champaign, IL, United States; ^4^The Graduate School, Purdue University, West Lafayette, IN, United States

**Keywords:** postdoc fellow, mentoring, faculty diversity, systemic change, faculty hiring

## Abstract

In this paper, we describe the model for faculty diversity developed as part of the Professorial Advancement Initiative (PAI) funded under the NSF AGEP program. The PAI, consisting of 12 of the 14 Big Ten Academic Alliance universities,^1^ had the goal of doubling the rate at which the universities hired tenure-track minoritized faculty, defined by National Science Foundation as African Americans, Hispanic/Latinx, Native Americans, and Pacific Islanders. This paper reviews the key programmatic elements of the PAI and discusses lessons learned and the practices developed that helped the Alliance achieve its faculty diversity goal.

## Introduction

The changing demographics in the United States provide compelling motivation to address the underrepresentation of faculty of color and ethnic minorities, especially in the science, technology, engineering, and math (STEM) fields. However, racial and ethnic inequalities and lack of faculty diversity in STEM fields at institutions of higher education persist as impediments (Yadav et al., 2020). A recent report by the Pew research center found that while 45% of the undergraduate students in the United States are racial or ethnic minorities, only 24% of the postsecondary faculty are nonwhite (Davis and Fry, 2019). Within STEM fields, these numbers are even more dire. As one study found, Black and Hispanic faculty representation in STEM fields range from 0.7 to 5.1% as compared to 4.2–15.1% in non-STEM fields (Li and Koedel, 2017). Even the latest data from the National Science Foundation (NSF) suggest that the number of African Americans, Latinx, Pacific Islanders, and Native Americans in STEM fields ranged from 0.5% (computer and information sciences) to 5.8% in (life sciences; National Science Foundation, 2019). A recent survey of over 7,000 professors at PhD granting institutions in the United States found that within STEM, social sciences, and the humanities median childhood income of those faculty is 23.7% higher than the general public and they are 25 times more likely to have a parent with a PhD. This has important implications for diversifying the professoriate and ensuring the need for institutions of higher education to examine how institutional structures perpetuate inequality, inhibit faculty diversity, and “sustain barriers that prevent minoritized individuals from gaining access to beneficial resources” (Griffin, 2020, p. 282).

Given that having teachers with a demographic similar to the students has been shown to have positive effects on students’ sense of belonging and motivation (Egalite and Kisida, 2018), a diverse professoriate can also increase recruitment and retention of students of color in STEM fields. Over the years, many universities have focused on increasing the number of students of color graduating from doctoral programs. These efforts have had a marginal impact. According to the latest survey of earned doctorates, from 2010 to 2019, the proportion of doctorates earned by Hispanic and Latinx students grew from 6 to 8%, while the proportion of doctorates earned by African American students increased from 6 to 7% (National Science Foundation, 2019). The survey also reported that 46% of all doctoral students go on to a postdoctoral position, which while down from 55% in 2010, still represents a significant portion of the post-graduation employment (National Science Foundation, 2019) and a major source of future faculty. Ironically, relatively little attention has been paid to effectively supporting their academic career aspirations. The National Academy of Sciences (2014) pointed out that there is a lack of comprehensive understanding about experiences of postdocs, in general, and even less about postdocs of color.

A recent survey of 7,603 postdocs from 351 institutions found that the majority of the postdocs (57.7%) saw academic research as a long-term goal with industry research as the distant second career goal (17.8%; McConnell et al., 2018, p. 9). The authors found that postdocs’ choice to pursue a research-focused academic career was positively correlated with postdocs’ views about mentoring support as well as their feelings of career preparedness. The authors further argue that an increase in “mentor support and mentorship may be a particularly important tool for increasing female and under-represented postdocs’ pursuit of research-intensive academic careers.”

At the same time, prior research has found that postdocs of color face “micro-aggressions, challenges to their competence, different work expectations, expectations to be representatives of minorities in general, and different treatment” (Yadav et al., 2020, p. 176). As a result, postdocs of color often do not have a sense of belonging and feel isolated at their institutions, which is further exacerbated by lack of professional development (Yadav et al., 2020). Yadav et al. (2020) argued that we need to move away from the “one size fits all” model of professional development (PD) and develop support systems specifically for scholars of color. In this paper, we report on the model developed as part of the Big Ten Academic Alliance PAI program.

## The Pai Model

To increase the representation of faculty of color, the PAI took a multi-pronged approach, which involved: (a) creating within the alliance a pool of postdocs of color who subsequently were mentored to enter the academy as tenure-track faculty members; (b) fostering systemic change in the faculty hiring processes to increase the diversity of the applicant pool, reduce the negative impacts of implicit bias in the selection process, and mitigate practices that unfairly favor applicants of the majority demographic.

### Postdoc and Faculty Mentor Professional Development

Our research identified a number of areas in which postdocs reported they needed mentoring to transition successfully to a tenure-track faculty role (Yadav and Seals, 2019). Most notably, postdocs reported that they needed support in developing their writing skills and ability to secure funding, which are important to be successful in a tenure-track position in our consortium of universities. In addition, postdocs reported a lack of sense of belongingness in their disciplines. In order to address these issues, we engaged postdocs and their faculty mentors in three primary activities: postdoc and faculty mentoring, cross-institutional webinars, and in-person workshops. These activities were informed by the needs of the postdocs that we identified by in-depth qualitative interviews conducted prior to the start of the workshops (see Yadav et al., 2020 about the postdoc needs) as well as suggestions from the postdocs as we did face-to-face and online workshops. Most importantly we discussed the role of mentors, sponsors, and coaches in career development.

The first step was the development of a postdoc mentoring guide for both postdocs and mentors that used the best practices from many sources including the National Postdoctoral Association, the National Institutes of Health, and the National Science Foundation. This guide was used by the mentee and mentor to formulate a customized Individual Development Plan for each postdoc in the PAI program. The purpose of the guide was to help facilitate conversations around PD needs and develop skills necessary for a faculty position. In addition to the mentoring guide, we also engaged both postdocs and mentors in PD activities.

The aim of the postdoc focused PD activities was to equip postdocs with important academic success skills, such as writing effective grant proposals, navigating the job application process, and creating a teaching and research plan. Results from our work suggested that the PD significantly improved postdocs’ self-efficacy across the grantsmanship skills and job application process (Yadav and Seals, 2019). Specifically, our results indicate that postdocs were significantly more confident in their grant writing skills after the PD (N=41, M=10.40, SD=1.95) than before (N=41, M=6.37, SD=2.30) as well as applying for academic jobs after the PD (N=41, M=20.00, SD=3.58) than before (N=41, M=14.62, SD=4.29; see Yadav and Seals, 2019 for detailed information about the impact of the workshops). The following quote from a PAI postdoc participant further highlights our impact, “Since being in this program [PAI], I received great mentorship – all aspects of the faculty application process – including the interview as well as what happens once I become a faculty member.”

While the function of these activities was to focus on specific skill building, an added value was the sense of community among the postdocs within their own universities as well as across the universities. In particular, the community building provided opportunities for minoritized postdocs to engage with other minoritized postdocs and share their experiences and perspectives as a person of color. For example, understanding the unwritten rules at a predominantly white institution (PWI) could be entirely different for a person of color. Over 200 postdocs participated in the PD that covered a wide range of topics (see Table 1 for a detailed description of the activities). In addition to these face-to-face PD activities, we also engaged postdocs in a series of webinars to support their transition into the academy. Examples of the webinars include how to publish in peer-reviewed journals, research-based strategies for overcoming imposter syndrome, and planning for a successful transition to a faculty position.

**Table 1:** Listing of the variety of PD workshops.

Activity	Description
Grantsmanship workshops	These workshops focused on how to find grants and collaborators, write grants, as well as understand the review process. Insights to the university process and a review panel discussion were given. The various parts of a grant were described and examples of successful applications were examined. Postdocs were able to discuss the process for cross disciplinary teams as well as project management and team success.
Academic hiring and job search workshop	This workshop included providing postdocs with the institution’s point of view for hiring faculty, how to prepare job application materials (e.g., cover letter, CV, teaching, research, and diversity statements), job interview process, and negotiating job offers.
Personal development and belongingness workshops	These workshops included discussion on how to navigate academic politics as a junior faculty member, especially at PWIs, and specifically designed to address the unique concerns of under-represented faculty members. The postdoc participants were engaged in the planning process by sending topics and themes they felt were important for their professional development. The speakers were intentional in expressing that we are giving them tools to help navigate a broken system that included hiring practices that the PAI project also worked to improve. Speakers were able to address the impact of being the “only one” in a department or team and how to navigate a culture that may have little knowledge of cross race mentoring.
Teaching workshop	This workshop focused on how to be an effective teacher, including developing competencies for teaching online. In addition, the activities also focused on what it means to teach as a faculty of color at a PWI.
Mentoring workshops	These workshops were specifically tailored to postdocs who are in the dual role of mentees to their PI and mentors-in-training as future faculty members. As such, the training provided a focus on both mentoring up and mentoring down. The mentoring workshops leveraged the work of Handelsman et al. (2005) to develop postdocs as effective mentors. Specifically, the workshops involved learning to communicate with mentees, setting goals and expectations, identifying and resolving challenges/issues, and developing aspects of good mentoring. We also addressed mentoring across cultures and finding mentors, sponsors, and coaches.

To further assist PAI postdocs in securing faculty positions, we created a directory that is designed to increase the visibility of our postdocs to faculty search committees. The searchable, online database is publicly accessible and includes relevant information about postdocs for search committees (e.g., education, research, and contact information). The Big Ten universities strongly encourage search committees to utilize this tool for recruitment with some even requiring search committees to actively recruit from the database. Since 2014, a total of 152 postdocs have opted into the directory with 62 transitioning to a faculty position and 22 into other positions within higher education.

### Systemic Change in Hiring Faculty

Complementing the PAI’s focus on postdoc mentoring and coaching was an effort to educate faculty about diversity and inclusion. An important part of this effort was engaging faculty members in an interactive workshop setting, where attendance by those serving on faculty search committees was required or strongly encouraged at each of the participating institutions. The general approach was borrowed from train-the-trainer workshops developed by the Women in Science and Engineering Leadership Institute at UW-Madison (WISELI),^2^ University of Washington ADVANCE program,^3^ and Purdue ADVANCE^4^ – programs aimed at women in STEM fields. The workshops benefited tremendously from the willingness of these program leaders to share their innovations and materials, which in turn allowed the PAI to develop workshop materials aimed at hiring minoritized scholars.

The first part of the workshop was devoted to establishing the compelling need to diversify the campus community and highlighting the academic benefits of educating students in an inclusive academic environment. We highlighted the importance of those involved in faculty hiring to recognize that diversity is essential, that it provides a competitive advantage, and that inclusion of diverse scholars must be a priority for universities.

A section of the workshop was devoted to “active recruiting” where we discuss aggressive recruiting strategies and the inadequacy of simply placing an ad to attract minoritized applicants. We have heard faculty say *qualified minority candidates do not exist in my field or are few and far between* as justification for why their applicant pool is not diverse. In the workshops, we discussed how to find and attract talented minoritized candidates to apply for faculty positions. An important tool in this regard is the postdoc directory mentioned earlier, which is now a national database. As universities across the country increase the number of URM PhDs they graduate, we expect the PAI database and others like it to grow in proportion.

After a discussion of strategies to achieve a diverse applicant pool, we turned attention to the selection process and practices often employed that unfairly disadvantage minoritized applicants. In support of maximizing the faculty interaction during the workshops, we developed a series of 5min videos depicting faculty search committee scenarios, each intended to set the stage for workshop discussion.

Three general sets of videos were produced. The first set includes three videos of a search committee just getting started. The second set consists of three videos depicting a search committee in the process of reviewing applications with the goal of narrowing the field to three finalists for campus interviews. The third set included four related videos of a committee in the final phase after campus interviews had occurred. The videos were designed to sharpen awareness among participants about practices and behaviors, particularly those that are subtle and unfairly disadvantage candidates from minoritized groups.

The workshops also included recommendations on communicating and interacting with candidates throughout the process and an overview of legal issues. Although the workshop is conservative from a legal perspective, we suggest that each university has its legal office review this part of the workshop content.

**Figure 1:**
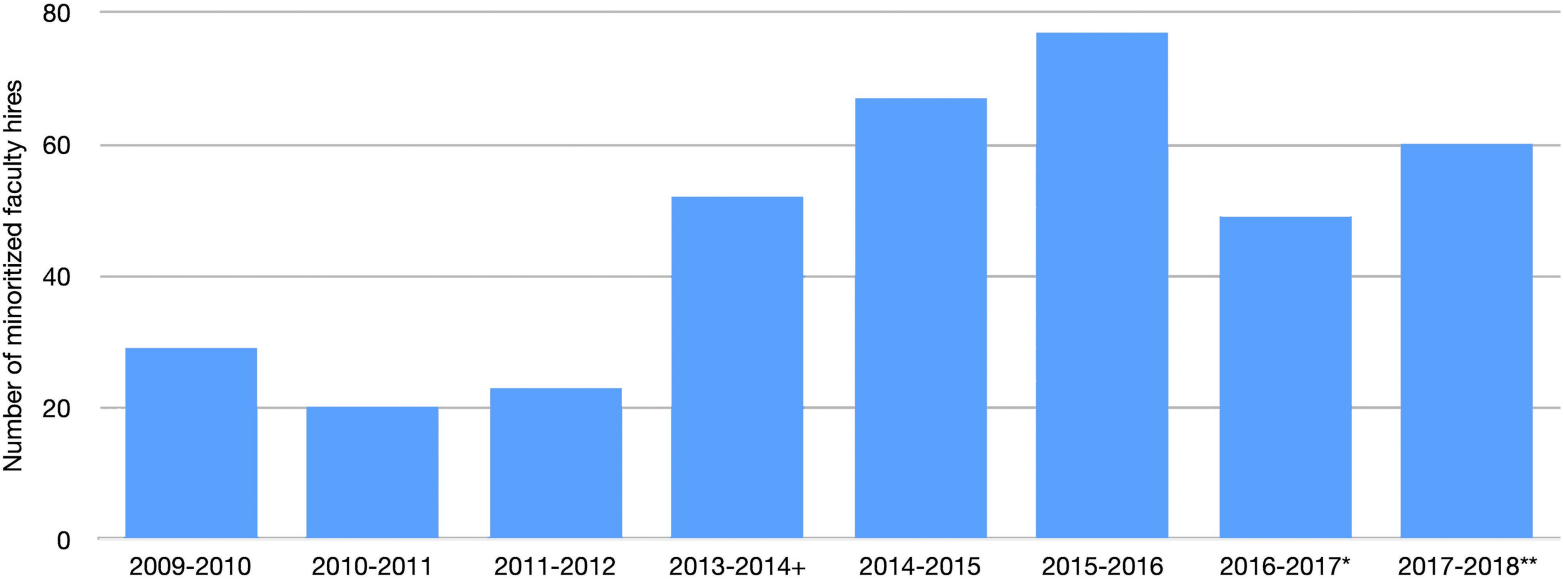
Minoritized faculty hiring across participating institutions. +First year of the project. *Data not available from two participating institutions. **Data not available from one participating institutions.

The efficacy of our model to increase diversity in the professoriate has been measured by the number of minoritized faculty hired during the program at the participating institutions as well as the number of minoritized postdocs who have successfully obtained faculty positions at other institutions. Figure 1 below shows the breakdown of the number of minoritized faculty hired before the start of our project in 2013. Since the start of the program, 312 minoritized faculty have been hired at participating universities since 2013 with the highest increase 2years after the program launched (2015–2016). Note that we did not get data from two institutions during the 2016–2017 academic year and data from one institution during 2018–2019year. In addition, 178 minoritized postdocs have participated in the program since 2013 from which 62 obtained faculty positions and 100 work within a university context, including as research scientists or in their current positions.

## Discussion

Over the years of offering the PAI workshops, we have gained many insights through the extensive discussions and information sharing that occurred during these meetings. Several of the most significant recommended practices and observations are mentioned next.

First, when the PAI faculty hiring workshops were first introduced, the notion of unconscious bias was not familiar to many if not most of the attendees. Now virtually everyone who attends the workshops has had some exposure to this topic, and many (if not most) have been involved in multiple diversity and inclusion seminars and workshops within their departments. Therefore, it is important during each offering of the workshop that current obstacles to hiring minoritized scholars are identified (through the workshop discussions) and then addressed in subsequent offerings. Related to this point, the workshops should include facilitated discussion, where everyone can contribute and learn from the experiences of others.

Another practice that has worked well is to have a facilitator at each table, who receives a short briefing handout ahead of time and can facilitate the discussion after viewing a video or session presentation. This allows the participants to stay focused on the topic and ensure that all major points are addressed. We invite the facilitators several times during the year to serve in this role. As they participate, they learn the format and material and are able to lead as session presenters in subsequent workshops.

In order to increase diversity in faculty hiring, we have also come to recognize the importance of establishing policies that support diverse hiring. A notable example of which is the university’s workshop attendance policy. When workshop attendance is on a voluntary basis, attendees who participate are typically those who are most informed about the issues. We have a greater impact when participation is strongly encouraged or mandated, particularly for search committee chairs. The greatest benefit is achieved when all search committee members have attended. Another policy that has been effective is to have an oversight mechanism in place to monitor the diversity of the applicant pool for each faculty search. If the pool is not diverse, the overseer (which could be a committee, chair, or dean) would stop the search and require the committee to start over.

When we looked at departments that were not diverse, it appeared faculty demographics were not seen as an urgent problem. A good way to bring attention to department diversity is to make it a topic of reflection and assessment during the annual review of department chairs by deans, and the annual review of the deans by the provost. Similarly, it can be helpful to have “contributions to diversity” in the context of research, teaching, and service as an item for reflection and consideration in the annual review of faculty and as part of the promotion and tenure process.

In summary, our model’s success indicates the importance of institutional commitment to increasing the number of faculty of color. One form of the commitment involves developing policies to change the hiring practices and educating faculty on how racial, ethnic, and gender biases can impact who gets interview opportunities for faculty positions and who eventually gets hired into those positions. At one of the participating institutions, policy change made it mandatory for those serving on search committees to attend faculty hiring workshops that discussed subtle bias using the videos discussed previously. In addition, the importance of this was highlighted by the fact that the Provost of the institution kicked off the workshops. Another aspect of the commitment involved each institution developing local capacity to deliver workshops and follow-up refresher courses. Our project team included trainers who visited each of the campuses and trained facilitators at each institution to deliver the faculty hiring workshops.

## Data Availability Statement

The original contributions presented in the study are included in the article/supplementary material, further inquiries can be directed to the corresponding authors.

## Author Contributions

AY, MS, CF, and LM contributed to conception of this paper. AY wrote the first draft of the manuscript. MS, CF, and LM wrote the sections of the manuscript. All authors contributed to the article and approved the submitted version.

## Funding

This work was funded by the National Science Foundation (NSF) under grants 1309028 and 1309173. The opinions expressed are those of the authors and do not necessarily reflect those of NSF.

## Conflict of Interest

The authors declare that the research was conducted in the absence of any commercial or financial relationships that could be construed as a potential conflict of interest.

## Publisher’s Note

All claims expressed in this article are solely those of the authors and do not necessarily represent those of their affiliated organizations, or those of the publisher, the editors and the reviewers. Any product that may be evaluated in this article, or claim that may be made by its manufacturer, is not guaranteed or endorsed by the publisher.

## Footnotes

^2^ https://wiseli.wisc.edu

^3^ https://advance.washington.edu

^4^ https://www.purdue.edu/advance-purdue/
